# Comparing the Survival Outcomes of Radical Prostatectomy *Versus* Radiotherapy for Patients With *De Novo* Metastasis Prostate Cancer: A Population-Based Study

**DOI:** 10.3389/fonc.2021.797462

**Published:** 2021-11-25

**Authors:** Xiaoxiao Guo, Haoran Xia, Xiaonan Su, Huiming Hou, Qiuzi Zhong, Jianye Wang

**Affiliations:** ^1^ Department of Urology, Beijing Hospital, National Center of Gerontology, Beijing, China; ^2^ Graduate School of Peking Union Medical College, Beijing, China; ^3^ Department of Radiotherapy, Beijing Hospital, National Center of Gerontology, Beijing, China; ^4^ Department of Urology, Zoucheng People’s Hospital, Zoucheng, China

**Keywords:** metastatic prostate cancer, propensity score analysis, radical prostatectomy, radiotherapy, comparative effectiveness

## Abstract

**Purpose:**

The efficacy of local treatments (LTs) in selected patients with metastatic prostate cancer (mPCa) had been demonstrated. However, the comparative effectiveness between LTs is unclear. Here, we compared the impact of radical prostatectomy (RP) and brachytherapy (RT) on the survival outcomes of mPCa patients.

**Materials and Methods:**

mPCa patients who received RT or RP between 2004 and 2016 were identified from the Surveillance, Epidemiology, and End Results (SEER) database. Multivariable Cox proportional hazards analysis was used to evaluate the comparative risk of prostate cancer-specific mortality (CSM) and all-cause mortality (ACM) between LTs. A 1:1 propensity score matching (PSM) and adjusted standardized mortality ratio weighting (SMRW) were performed to balance the clinicopathological characteristics of the groups.

**Results:**

Of 684 mPCa patients, 481 underwent RP and 203 received RT. After PSM, both groups included 148 cases, and RT resulted in comparable CSM *versus* RP [CSM: hazard ratio (HR) = 0.77, *p* = 0.325; ACM: HR = 0.73, *p* = 0.138], which was consistent with the SMRW model [CSM: HR = 0.83, *p* = 0.138; overall survival (OS): HR = 0.75, *p* = 0.132]. However, RP was associated with a lower CSM in the T_1–2_ subgroup (HR = 0.42, *p* = 0.048) and a lower ACM in the T_1–2_ (HR = 0.55, *p* = 0.031) and prostate-specific antigen (PSA) ≤20ng/ml (HR = 0.48, *p* = 0.022) subgroups. Besides, the results showed that the mortality risk was similar between the two groups in the T_3–4_, Gleason score (GS) >7, PSA >20 ng/ml, and all metastatic subgroups (all *p* > 0.100).

**Conclusions:**

RP could confer better survival outcomes than could RT in mPCa patients with favorable primary tumor features, but not in those with advanced primary tumor features. Moreover, the metastatic substage has limited impact on the comparative effectiveness between RP and RT. Further clinical trials are necessary to confirm the present results.

## Introduction

Recently, the landscape of the management of metastatic prostate cancer (mPCa) is changing rapidly with the new generation of hormone therapies and other treatment options emerging and changing the paradigms that have persisted for decades (1). In this context, there is growing interest in whether local treatments (LTs) for the primary lesion could improve the survival outcomes of mPCa patients. This hypothesis is based on the seed and soil theory wherein, in animal models of cancer, the primary tumors metastasize not only by disseminating tumor cells into circulation but also by preparing the so-called pre-metastatic niche for metastasis implantation (2). The proliferation of metastasis at distant sites is stimulated and maintained by compounds secreted by primary cancer into circulation (3). Based on these concepts, LTs of the primary tumor could improve survival through inhibiting not just the initiation of distant disease but also the progression of the existing metastases.

Previous population-based studies had postulated a survival benefit for LTs *versus* non-local treatments (NLTs) in *de novo* mPCa patients (4–9). Furthermore, prospective randomized studies addressing the effect of LT by radiotherapy on the outcome of mPCa patients demonstrated its effect on selected individuals (10, 11). However, the comparative effectiveness between LTs, such as radical prostatectomy (RP) and brachytherapy (RT), is unclear. To this aim, we compared the impacts of RP and RT on the survival outcomes after adjustment for other clinicopathological characteristics in the most contemporary population-based cohort of mPCa patients.

## Patients and Methods

### Study Population

We used the Surveillance, Epidemiology, and End Results (SEER) database to identify patients with mPCa from 2004 to 2016. The 18th SEER tumor registries encompass ~26% of the US population and collect information on cancer incidence, prevalence, survival, and mortality of cancer patients.

**Figure 1:**
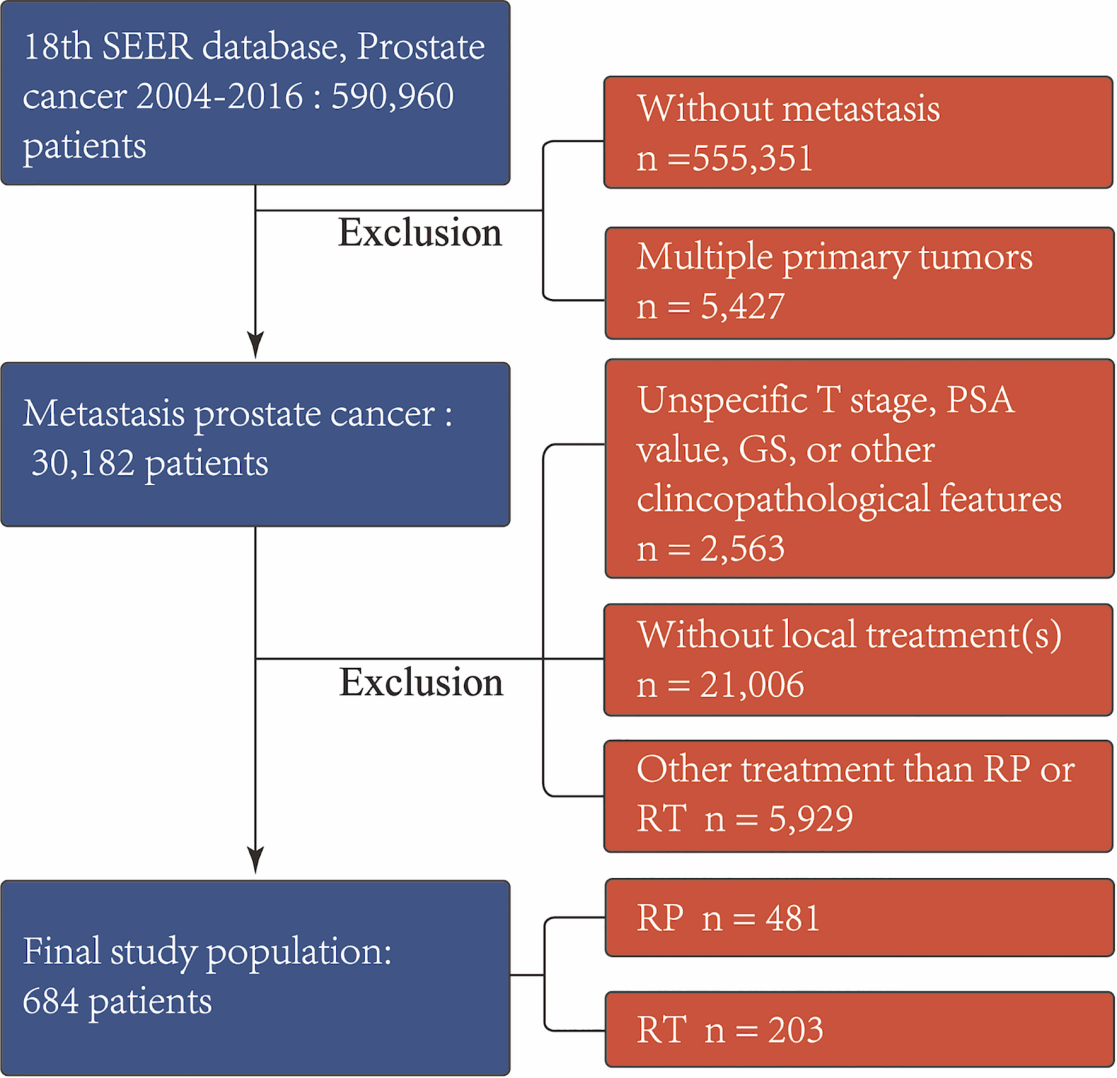
Exclusion criteria used to derive the final study cohort from the SEER database from 2004 to 2016. *RP*, radical prostatectomy with or without external beam radiation therapy; *RT*, brachytherapy with or without external beam radiation therapy; *PSA*, prostate-specific antigen; *GS*, Gleason score.

Firstly, we identified 590,960 patients diagnosed with prostate cancer (PCa). Individuals who underwent 1) RP with or without external beam radiation therapy or 2) RT with or without external beam radiation therapy were included. We excluded those who were without metastasis or had multiple primary tumors. Then, patients with incomplete clinicopathological data, such as T stage, prostate-specific antigen (PSA) value, and Gleason score (GS), were excluded. Those who received external beam radiation therapy alone were also excluded since there was lack of target site information distinguishing local from extraprostatic treatment. In addition, cases who received surgical treatments other than RP, e.g., transurethral resection of the prostate or cryotherapy, were removed. These selection criteria yielded 684 patients. The patient inclusion and exclusion diagrams are depicted in **Figure 1**.

### Propensity Score Matching

Propensity score matching (PSM; a 1:1 matching algorithm with a specified caliper distance of 0.001 of the standard deviation of the logit) was conducted to yield similar patient characteristics between the radiotherapy (*n* = 148) and RP (*n* = 148) cohorts, imitate a randomized trial design, diminish residual and selection bias, and enhance precision. Age, year of diagnosis, the PSA level, clinical tumor stage, biopsy GS, and the American Joint Committee on Cancer (AJCC) M stage were adjusted using the logistic regression model to calculate the propensity scores. The standardized mean difference (SMD) and a propensity score density plot were used to evaluate the matching efficiency.

### Statistical Analysis

A comparison of the baseline characteristics between the RT and RP groups was conducted for the unmatched and PSM cohorts using *χ* ^2^ and Fisher’s exact tests. Metastatic stages (M-stages) are sub-stratified into the following: M1a, “non regional lymph nodes”; M1b, “bone(s)”; and M1c, “other site(s) with or without bone disease. Hazard ratios (HRs) with 95% confidence intervals (95%CIs) for all-cause mortality (ACM) and cancer-specific mortality (CSM) owing to RT *versus* RP were calculated using the multivariate Cox proportional hazards model, both in the unmatched and PSM cohorts, with age, PSA, race, biopsy Gleason score, clinical tumor stage, and metastatic substages serving as covariates. The standardized mortality ratio weighting (SMRW) model that makes the distribution of risk factors equal to that of the RT group was used to confirm the robustness of our outcomes. The overall survival (OS) and cancer-specific survival (CSS) of patients treated with RT or RP in the unmatched and PSM cohort were estimated using the Kaplan–Meier survival curve. Subsequently, subgroup analyses on the basis of clinical tumor stage (T_1–2_ and T_3–4_), metastatic substage (M_1a–1b_ and M_1c_), biopsy GS (≤7 and >7), and PSA levels (≤20 and <20 ng/ml) were conducted. All tests were two sided, with statistical significance set at *p* < 0.05. Analyses were performed with the statistical package for R (version 3.6.1; the R Foundation for Statistical Computing, Vienna, Austria).

## Results

**Figure 2:**
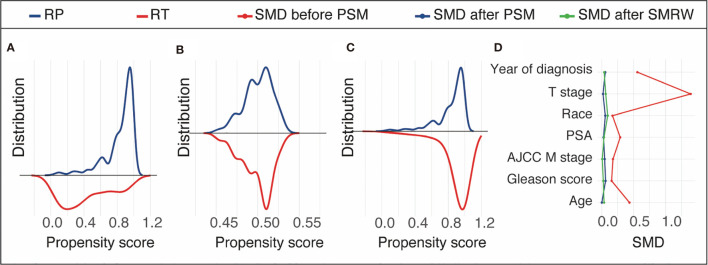
Propensity score distribution in the unmatched cohort **(A)**, propensity score matching (PSM) cohort **(B)**, and the standardized mortality ratio weighting (SMRW) cohort **(C)** and the standardized mean difference (SMD) of the key variables in the unmatched, PSM, and SMRW cohorts **(D)**. *RT*, brachytherapy with or without external beam radiation therapy; *RP*, radical prostatectomy with or without external beam radiation therapy.

As **Table 1** shows, prior to PSM, the RT and RP groups included 203 and 481 patients, respectively. Patients in the RT group were older (68 years) relative to those in the RP group (63 years). The rate for clinical tumor stage T2 or lower was higher in the RT group (83.2%) than that in the RP group (34.9%). The rate for Gleason score ≥7 was higher in the RP group (90.9%). Patients who underwent RP had a larger proportion of M_1a_ disease than those treated with RT (17.3% *vs*. 8.9%). Besides, those in the RP group have been generally treated more recently (*p* < 0.001) compared with those in the RT group. The two groups were well balanced after PSM, given that no significant differences were observed for all key variables. The propensity score distributions of the RT and RP groups in the unmatched cohort was highly heterpgenerious (**Figure 2A**) and it in the PSM and SMRW cohorts were highly consistent (**Figures 2B, C**). All of the SMD values of the key variables in the PSM and SMRW cohorts were <10% and were far less than those in the unmatched cohort (**Figure 2D**).

**Table 1:** Baseline demographic and clinicopathologic characteristics sorted by local treatments before and after PSM.

	Before PSM			After PSM		
	RT (*n* = 203)	RP (*n* = 481)	*p*-value	RT (*n* = 148)	RP (*n* = 148)	*p*-value
Age (year)^a^	68.0 (59.5–73.0)	63.0 (57.0–67.0)	<0.001	65.0 (56.0–72.0)	65.0 (58.0–69.0)	0.208
PSA (ng/ml)^a^	15.5 (6.6–72.0)	9.9 (6.2–21.8)	<0.001	15.0 (5.8–38.7)	16.6 (6.3–31.0)	0.688
Race^b^			0.014			0.655
Caucasian	148 (72.9)	386 (80.2)		110 (74.3)	111 (75.0)	
African	44 (21.7)	62 (12.9)		24 (16.2)	27 (18.2)	
Other	11 (5.4)	33 (6.9)		14 (9.5)	10 (6.8)	
Year of diagnosis^b^			<0.001			0.548
2004–2007	80 (39.4)	100 (20.8)		58 (39.2)	36 (14.3)	
2008–2011	63 (31.0)	120 (24.9)		38 (25.7)	35 (23.6)	
2012–2016	60 (29.6)	261 (54.3)		52 (35.1)	57 (38.5)	
Clinical T stage^b^			<0.001			0.107
T_1_	102 (50.2)	14 (2.9)		18 (12.2)	8 (5.4)	
T_2_	67 (33.0)	154 (32.0)		72 (48.6)	86 (58.1)	
T_3_	27 (13.3)	267 (55.5)		48 (32.4)	48 (32.4)	
T_4_	7 (3.4)	46 (9.6)		10 (6.8)	6 (4.1)	
Gleason score^b^			0.014			0.851
≤6	33 (16.3)	44 (9.1)		18 (12.2)	15 (10.1)	
7	55 (27.1)	164 (34.1)		40 (27.0)	40 (27.0)	
≥8	115 (56.7)	273 (56.8)		90 (60.8)	93 (62.8)	
AJCC M stage^b^			0.009			0.928
M_1a_	18 (8.9)	83 (17.3)		24 (16.2)	26 (17.6)	
M_1b_	145 (71.4)	328 (68.2)		94 (63.5)	94 (63.5)	
M_1c_	40 (19.7)	70 (14.6)		30 (20.3)	28 (18.9)	
Follow-up (months)^a^	45.0 (17.5–88.5)	34.0 (14.0–71.0)	0.01	56.5 (18.0–110.0)	37.0 (14.0–83.5)	0.013

PSM, propensity score matching; RP, radical prostatectomy with or without external beam radiation therapy; RT, brachytherapy with or without external beam radiation therapy; PSA, prostate-specific antigen.

a Data are presented as median (interquartile range).

b Data are presented as n (%).

**Figure 3:**
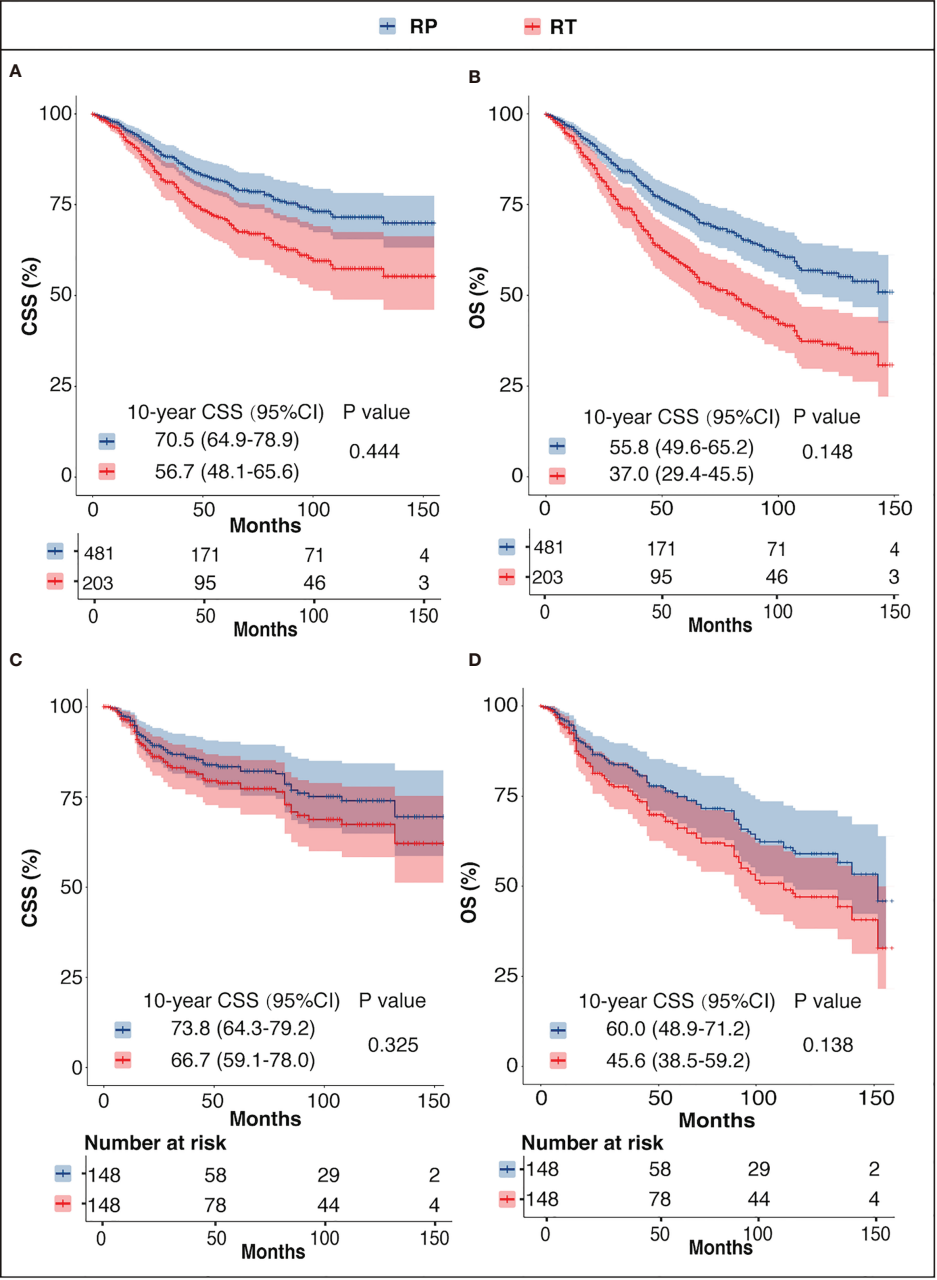
CSS and OS of the RP and RT groups before PSM **(A, B)** and after PSM **(C, D)**. *CSS*, prostate cancer-specific survival; *OS*, overall survival; *RP*, radical prostatectomy with or without external beam radiation therapy; *RT*, brachytherapy with or without external beam radiation therapy.

In the unmatched cohort, the Cox proportional hazards model revealed that there was no significant difference between the CSM or the ACM of the two groups (CSM: HR = 0.86, 95%CI = 0.57–1.27, *p* = 0.444; ACM: HR = 0.78, 95%CI = 0.56–1.09, *p* = 0.148). Consistently, in the PSM cohort, there was no significant difference regarding CSM or ACM risk between the two groups (CSM: HR = 0.77, 95%CI = 0.46–1.30, *p* = 0.325; ACM: HR = 0.73, 95%CI = 0.48–111, *p* = 0.138), which was supported by the outcomes in the adjusted SMRW model as well (CSM: HR = 0.83, 95%CI = 0.53–1.32, *p* = 0.435; ACM: HR = 0.75, 95%CI = 0.52–1.09, *p* = 0.123). The above results are summarized in **Table 2**. The Kaplan–Meier survival curves showed that the 10-year survival rates of RP *versus* RT before PSM were 70.5% *vs*. 56.7% for CSS and 55.8% *vs*. 37.0% for OS. After PSM, the 10 year CSS rates of RT *versus* RT were 73.8% *vs*. 66.7% and the 10-year OS rates were 60.8% *vs*. 45.6.0% (**Figure 3**) .

**Table 2:** Effect of RT and RP on the survival outcomes.

	HR	95%CI	*p*-value
Cancer-specific mortality			
Adjusted non-matched cohort	0.86	0.58–1.27	0.444
Adjusted PSM cohort	0.77	0.46–1.30	0.325
Adjusted SMRW cohort	0.83	0.53–1.32	0.435
All-cause mortality			
Adjusted non-matched cohort	0.78	0.56–1.09	0.148
Adjusted PSM cohort	0.73	0.48–1.11	0.138
Adjusted SMRW cohort	0.75	0.52–1.09	0.132

RP, radical prostatectomy with or without external beam radiation therapy; RT, brachytherapy with or without external beam radiation therapy; HR, hazard ratio; CI, confidence interval; PSM, propensity score matching; SMRW, standardized mortality ratio weighting.

**Figure 4:**
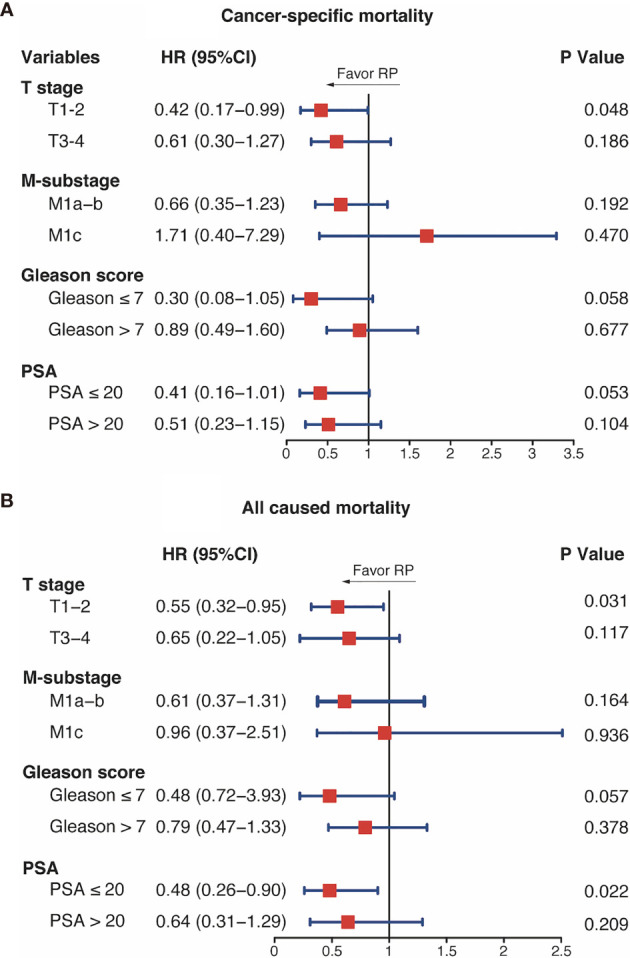
Subgroup analysis of cancer-specific mortality **(A)** and all-cause mortality **(B)** with brachytherapy (RT) and radical prostatectomy (RP). *HR*, hazard ratio; *CI*, confidence intervals; *PSA*, prostate-specific antigen; *GS*, Gleason score.

In the subgroup analysis, RP was associated with a lower CSM in the T_1–2_ subgroup (HR = 0.42, 95%CI = 0.17–0.99, *p* = 0.048). In addition, the CSM differences between RT and RP were close to being statistically significant in the GS ≤7 and PSA ≤20 ng/ml subgroups (HR = 0.30, 95%CI = 0.08–1.05, *p* = 0.058; HR = 0.41, 95%CI = 0.16–1.01, *p* = 0.053, respectively). However, the analysis showed that the CSM rates were similar between the two groups in the T_3–4_, GS >7, PSA >20 ng/ml, and all M-stage subgroups (**Figure 4**). Regarding the ACM, the RP group had better performance in the T_1–2_ and PSA ≤20 ng/ml subgroups (T_1–2_: HR = 0.55, 95%CI = 0.32–0.95, *p* = 0.031; PSA ≤20 ng/ml: HR = 0.48, 95%CI = 0.36–0.90, *p* = 0.022). In patients with GS ≤7 disease, the RP group had a tendency of lower ACM (HR = 0.079, 95%CI = 0.47–1.33, *p* = 0.057). Furthermore, RT and RP performed equivalently in patients in the T_3–4_, GS >7, PSA >20 ng/ml, and all M-stage subgroups.

## Discussion

So far, retrospective data from a few studies had demonstrated a survival benefit or reduction in locoregional complication by the addition of LT to androgen deprivation therapy (ADT) in men with *de novo* mPCa (6, 9, 12–16). Local treatment of a primary tumor might prove beneficial through altering the natural course of the disease by slowing down the progression of metastasis, as others have described that the untreated primary PCa may act as a potential source of tumor spread and metastasis (17, 18). Moreover, in patients with locally advanced or nodal positive PCa, it may also improve the response to ADT (19, 20). Furthermore, the improved local tumor control could thereby decrease the need for subsequent palliative therapies, such as transurethral resection or urinary diversion. However, contemporary data are lacking to recommend one alternative local therapy over another. In the present study, we compared the survival outcomes between RT and RP for mPCa patients using a method with rigid case matching.

In our analysis, for the total cohort, there were no statistical CSS or OS differences between the RP and RT groups. The results after PSM and SMRW analysis confirmed the comparable survival outcomes of the two treatments. However, these results were different from an earlier database-based study. Leyh-Bannurah et al. identified 313 patients who received radical prostatectomy and 161 patients who received radiotherapy in the SEER database (4). They also performed PSM for the two treatment groups. The results suggested that RP was associated with a lower CSM than was RT (HR = 0.59, *p* = 0.048). A potential reason for the different findings between our study and that of Leyh-Bannurah et al. is that, in the latter cohort, patients with favorable primary tumor features, such as T_1–2_ or GS ≤7, represented a higher proportion of all cases. In addition, RP had better performance in mPCa patients with favorable primary tumor features, which would be discussed later. Moreover, in another retrospective study, Satkunasivam et al. identified 47, 88, and 107 patients who underwent RP, intensity-modulated radiation therapy (IMRT), and conformal radiation therapy (CRT), respectively, from the SEER-Medicare database to assess survival after RP, IMRT, or CRT *vs*. NLTs for mPCa. After PSM, they found that IMRT (62%, *p* < 0.001) and RP (52%, *p* = 0.01) lowered the CSM, whereas CRT did not (*p* = 0.3), when compared to NLTs (4). These results suggested that various external radiotherapy techniques may be another reason for the differences in the conclusions between studies. However, the specific radiotherapy technique was unavailable in SEER; therefore, we cannot conduct further analysis.

The subgroup analysis showed that RP confers a better survival benefit than does RT in patients with cT_1–2_ disease. In addition, RP showed a tendency of more effectiveness in the Gleason ≤7 and PSA ≤20 ng/ml substages. A survival benefit, however, was not observed in patients with advanced primary tumor. One possible reason accounting for the similar outcomes between RP and RT in patients with advanced clinicopathological features is that, LTs, such as RP and RT, have little effect on survival for this population. As the results of a previously mentioned study reported (4), the effect of LTs on survival had an interaction with the risk criteria (GS ≥8, cT_4_, and M_1b–c_ substages); that is, RP and RT are less effective in patients with two or more risk criteria than in those with one or fewer risk criteria. Moreover, in another SEER-based study evaluating LTs for patients with mPCa, Pompe et al. found a significant interaction between PSA and treatment type in M1b patients. LTs conferred a survival benefit when PSA was <60 ng/ml, with maximum benefit when PSA was <40 ng/ml. However, no survival benefit existed for M1b patients above the 60-ng/ml PSA threshold (21). Furthermore, the results of the HORRAD trial also suggested the dissatisfactory efficacy of LTs in mPCa patients with advanced primary tumor characteristics. It randomly recruited 432 patients with PSA >20 ng/ml and primary bone mPCa between 2004 and 2014. The study demonstrated that the protective association of RP with CSM was not recorded in these individuals (11). These results noted that comparing the effectiveness of LTs must be conditional upon LTs being better than NLTs. If not, it is meaningless to compare the efficacy between LTs.

Our results suggested that M-substages has an insignificant influence on the comparative CSM risk of RP *vs*. RT (*p* = 0.192 in M_1a–b_ substages; *p* = 0.470 in M_1c_ substage). A handle of studies had evaluated the effect of M-substage on the efficacy of LTs. For example, Pompe et al. evaluated the impact of baseline PSA and metastatic substages on the survival benefit of LTs *vs*. NLTs for mPCa based on SEER and found that a survival benefit existed for patients with M_1b_ PCa, but not for those with M_1c_ PCa (21). Furthermore, the follow-up of the STAMPEDE trial suggested that the RT for mPCa did not confer an OS advantage for the complete study population, but did for the low metastatic burden subgroup, which was defined using CHAARTED (Chemohormonal Therapy *Versus* Androgen Ablation Randomized Trial for Extensive Disease in Prostate Cancer) (10). However, none of these studies was tailored to determine the effect of M-substage on the comparative effectiveness of RT and RP. Our results indicated that the efficacy of the different LTs is largely determined by the characteristics of the primary tumor rather than the M-substage.

Our study is not devoid of limitations. Firstly, site-specific external beam radiation therapy (EBRT) codes are unavailable in the SEER database and only RT codes were analyzed. Secondly, the SEER database does not identify additional therapies such as ADT, chemotherapy, or other systemic agents as they represent the gold standard therapy for men with mPCa (1). Thirdly, the SEER database does not provide complete information regarding the exact metastatic burden, such as the number of metastatic diseases, except for the M-substages that were included in the analyses. Finally, similar to all previous studies, this study is retrospective in nature. Besides, the SEER database does not provide information on comorbidities and performance status, which may be used for patient selection.

## Conclusions

Our study provides evidence that, for mPCa, RP could confer better survival outcomes than could RT in those with favorable local stage, Gleason score, or PSA value. However, in patients with advanced local tumor characteristics, RP and RT provide similar survival outcomes. Furthermore, the metastatic substage has a limited impact on the comparative effectiveness between RP and RT. It is important to consider the study limitations until ongoing clinical trials confirm the proposed benefits.

## Data Availability Statement

Publicly available datasets were analyzed in this study. This data can be found here: https://seer.cancer.gov/data/.

## Ethics Statement

The data in this study were derived from the SEER database. All procedures performed in studies involving human participants were in accordance with the ethical standards of the institutional and national research committee and with the 1964 Helsinki Declaration and its later amendments or comparable ethical standards. The SEER Program collects data from population-based cancer registries with anonymous information. It is a publicly available database; thus, no ethical approval is required.

## Author Contributions

XG and HX performed formal analysis and wrote the original draft. XS and HH helped with the methodology. JW conceptualized the study. QZ contributed to the conceptualization, funding acquisition, resources, and supervision. All authors contributed to the article and approved the submitted version.

## Funding

This study was supported by the Beijing Hospital Clinical Research 121 Project (BJ-2020-171).

## Conflict of Interest

The authors declare that the research was conducted in the absence of any commercial or financial relationships that could be construed as a potential conflict of interest.

## Publisher’s Note

All claims expressed in this article are solely those of the authors and do not necessarily represent those of their affiliated organizations, or those of the publisher, the editors and the reviewers. Any product that may be evaluated in this article, or claim that may be made by its manufacturer, is not guaranteed or endorsed by the publisher.
